# Self-assembly of a barnacle cement protein into intertwined amyloid fibres and determination of their adhesive and viscoelastic properties

**DOI:** 10.1098/rsif.2023.0332

**Published:** 2023-08-09

**Authors:** Maura A. Tilbury, Thi Quynh Tran, Dilip Shingare, Mathilde Lefevre, Anne Marie Power, Philippe Leclère, J. Gerard Wall

**Affiliations:** ^1^ Microbiology, School of Biological and Chemical Sciences, University of Galway, Galway, Ireland; ^2^ SFI Centre for Medical Devices (CÚRAM), University of Galway, Galway, Ireland; ^3^ Ryan Institute, School of Natural Sciences, University of Galway, Galway, Ireland; ^4^ Laboratory for Physics of Nanomaterials and Energy, Research Institute for Materials, University of Mons, 7000 Mons, Belgium; ^5^ Laboratory of Cell Biology, Research Institute for Biosciences, University of Mons, Place du Parc 23, 7000 Mons, Belgium

**Keywords:** protein adhesion, barnacle cement protein, viscoelastic nanoscale properties, amyloid fibre, self-assembly

## Abstract

The stalked barnacle *Pollicipes pollicipes* uses a multi-protein cement to adhere to highly varied substrates in marine environments. We investigated the morphology and adhesiveness of a component 19 kDa protein in barnacle cement gland- and seawater-like conditions, using transmission electron microscopy and state-of-the art scanning probe techniques. The protein formed amyloid fibres after 5 days in gland-like but not seawater conditions. After 7–11 days, the fibres self-assembled under gland-like conditions into large intertwined fibrils of up to 10 µm in length and 200 nm in height, with a distinctive twisting of fibrils evident after 11 days. Atomic force microscopy (AFM)-nanodynamic mechanical analysis of the protein in wet conditions determined *E*′ (elasticity), *E*′′ (viscosity) and tan δ values of 2.8 MPa, 1.2 MPa and 0.37, respectively, indicating that the protein is a soft and viscoelastic material, while the adhesiveness of the unassembled protein and assembled fibres, measured using peak force quantitative nanomechanical mapping, was comparable to that of the commercial adhesive Cell-Tak™. The study provides a comprehensive insight into the nanomechanical and viscoelastic properties of the barnacle cement protein and its self-assembled fibres under native-like conditions and may have application in the design of amyloid fibril-based biomaterials or bioadhesives.

## 1. Introduction

Barnacles are known for their adhesive abilities and are the only sessile members of the Crustacea who commit as larvae to settling on one spot, which will become their permanent home [1]. After metamorphosis, they permanently bind their base to an underwater substrate using cement, which mainly comprises multi-protein complex that is synthesized in a series of ‘giant’ cells within the peduncle (stalk) in stalked barnacles and in the basis beneath the mantle cavity in acorn barnacles [1–3]. Each giant adhesive cell is drained by a canal that delivers the liquid adhesive to the substratum, where it cures into a solid rubbery cement [2,4].

The underwater adhesion process is proposed to involve surface functions, such as displacement of the water layer and coupling of the adhesive to the surface, and bulk functions, such as curing to provide stiffness, strengthening of the adhesive and protection from microbial degradation [5]. The constituents of the adhesive complex have potential applications in biotechnology and biomedicine, as seen with proteins from marine organisms such as flatworms [6], mussels [7–11], sandcastle worms [12,13] and starfish [14,15]. The adhesive novelties associated with barnacles are suggested to be self-assembling peptides based on non-covalent interactions [3], with assembly leading to nanofibril formation [16] via processes that are potentially tunable according to their environment.

Six barnacle cement proteins (cp) have been characterized to date, of which the insoluble 100 and 52 kDa cps are thought to provide bulk properties in the barnacle cement, a 16 kDa cp has been suggested to be a lysozyme-like enzyme involved in surface preparation, and the 19, 20 and 68 kDa cps have been proposed to be more adhesive and provide surface functions [3,17]. cp19 in particular, upon first identification from *Megalabanus rosa*, was proposed to function in coupling to foreign material surfaces during underwater attachment [18]. An acceleration of high-throughput omics analyses in the past 10 years has led to the identification of many more proteins in basal tissue transcriptomes that are potentially active at the adhesive interface in barnacles [19,20].

*Pollicipes pollicipes* is a large stalked barnacle with a membranous base, which cements to intertidal rocks using an adhesive formed within its cement gland cells, makes its way in liquid form through a series of canals and is drained to the rocky substratum via a pair of principal canals, where it hardens into a white rubbery solid [21]. Adults of this species can move slightly [22], possibly enabled by the membraneous base [23]. Recently, full gene sequences for *P. pollicipes* cement proteins cp19-K, cp52-K and cp100-K, named for their predicted respective molecular weights of 19, 52 and 100 kDa, have been verified [24], along with an assembled transcriptome of *P. pollicipes* adhesive glands [25] and proteomic analyses [20,24,26]. However, the functional characterization of stalked barnacles has mainly been limited to *in silico* analyses to date [19,24].

We previously carried out heterologous expression and purification of *P. pollicipes* 19 kDa cp (rPpolcp19k), followed by adhesion assays on differing surface chemistries [27]. The protein, though a homologue of the surface-coupling type from acorn barnacles [3,18], was not more adhesive than controls under conditions that mimicked seawater and gland conditions. Instead, it was speculated to form functional amyloids based on its amino acid profile [27]. Functional amyloids, so-called to distinguish their beneficial functionality from plaque-forming amyloids associated with diseases such as Alzheimer's and Parkinson's disease, are characterized by self-assembly into fibrils [16,28–31], mediated by a unique cross β-sheet structure [32,33].

Numerous studies of the nanostructure and mechanical properties of amyloid fibrils have been carried out under liquid conditions using atomic force microscopy (AFM) approaches such as force volume (FV), peak force quantitative nanomechanical mapping (PFQNM) and quantitative imaging (QI). These typically investigate elasticity and adhesion but overlook viscosity, which plays a vital role in the surface coverage and wetting ability of a protein [34]. AFM-nanodynamic mechanical analysis (AFM-nDMA) was developed in 2019 and allows the measurement of detailed viscoelastic properties of fibres in native-like conditions at the low rheological frequency range of 0.1 to 100 Hz [35,36]. The approach takes advantage of extended tip-sample contact times compared with FV, PFQNM or QI AFM modes, allowing the tip to be oscillated at a specific frequency on the sample surface, and the amplitude and phase of the cantilever deflection and Z-piezo motion to be collected for determination of the storage modulus (*E*′—the ability of material to store energy elastically), loss modulus (*E*′′— the ability of the material to dissipate energy) and loss tangent (tan δ—the ratio between the storage and loss moduli) of a material.

The present paper describes the formation of amyloid fibrils from rPpolcp19k, establishes the conditions in which they are produced and uses AFM-nDMA to examine their morphologic and viscoelastic nanoscale properties and their self-assembly, under both air and liquid conditions.

## 2. Material and methods

### 2.1. Recombinant protein expression and purification

rPpolcp19k-his protein was expressed in *Escherichia coli* BL21 (DE3) cells and purified by immobilized metal affinity chromatography (IMAC), with column washing carried out using 200 mM sodium phosphate buffer containing 60 mM imidazole, and elution using the same buffer containing 150 mM imidazole. This was followed by analysis by SDS-PAGE and immunoblotting, as previously described [27].

### 2.2. Self-assembly assay

Following buffer exchange using Zeba Spin Desalting Columns (Thermo Fisher Scientific), 500 µg ml^−1^ rPpolcp19k-his was re-constituted in 10 mM sodium acetate buffer (pH 4.0) containing 150 mM NaCl (designed to mimic the barnacle cement gland; [1,37]), or 10 mM sodium phosphate buffer (pH 8.0) containing 600 mM NaCl, (designed to mimic seawater) and incubated at 25°C for up to 11 days. rPpolcp19k-his samples were removed at intervals, snap frozen in liquid nitrogen and stored at −80°C for further analysis.

### 2.3. Thioflavin T assay

To investigate the self-assembly of rPpolcp19k into amyloid fibres, rPpolcp19k-his (0.5 mg ml^−1^) was incubated at 25°C in the barnacle cement gland-like or seawater-like conditions described above. Samples taken after 0, 5, 7 and 11 days were assessed for the presence of amyloid using Thioflavin T (ThT), which displays enhanced fluorescence and a red-shift in its emission spectrum upon binding to the β-sheet-rich regions of amyloid fibrils [38]. cp19k samples (100 µl) were mixed with a final concentration of 20 µM ThT and incubated at room temperature for 5 min. Fluorescence emission was measured at 482 nm in a black polystyrene 96-well plate in a Varioskan Flash spectrofluorometer, at 440 nm excitation and 12 nm bandwidth. Spectra were plotted after subtraction of buffer fluorescence values.

### 2.4. Transmission electron microscopy

5–10 µl of rPpolcp19k-his protein samples (500 µg ml^−1^), taken after 0, 5, 7 or 11 days' incubation under gland- or seawater-like conditions, were loaded onto a 200 mesh Cu formvar/carbon grid (Agar Scientific) and allowed to settle for 5 min. Samples were washed three times in buffer for 5 min, followed by three washes in dH_2_O. Samples were negatively stained by incubating 5 µl of 2% (w/v) uranyl acetate solution on the grid for 3 min. Grids were washed five times with dH_2_O and dried overnight. Bright-field transmission electron microscopy (TEM) images at different magnifications were collected on a Hitachi H7000 transmission electron microscope operated at an accelerating voltage of 80 kV.

### 2.5. Atomic force microscopy

Atomic force microscopy (AFM) was first carried out in tapping mode (TM-AFM) to study the topography of proteins at high resolution, followed by PFQNM and AFM-nDMA in liquid to investigate their mechanical (elastic and viscoelastic) properties. Samples were prepared by placing a 40 µl drop of barnacle cement proteins on the appropriate glass surface pre-cleaned using 5% HCl. For measurements in air, samples were placed on microscope coverslips and allowed to dry overnight in a fume hood. For measurements in liquid, samples were prepared on microscope slides and kept in a humid environment at room temperature for 16–18 h, followed by gentle washing three times with Milli Q water.

All AFM measurements were performed at 25°C using a Dimension Icon AFM (Bruker) and Nanoscope software v. 9.7. TM-AFM was performed in air and used a TESPA-50 probe (Nanoworld, NanoAndMore GMbH) with a radius under 8 nm, 42 N m^−1^ spring constant and 320 kHz resonance frequency. The cantilever was oscillated at its resonant frequency (320 kHz for TESPA-50) and samples were mapped at 512 × 512 pixels per image.

Force spectroscopy (force modulation) measurements were made in air using PFQNM with a RTESPA-150–30 probe (Bruker AFM Probes, Camarillo, CA, USA) pre-calibrated with a 5 N m^−1^ spring constant and a tip radius of 30 nm. The probe was calibrated on sapphire, allowing conversion of the photodiode signal (in volts) to deflection of the cantilever (in nm), while mechanical properties were calculated by fitting the approach or retract curve corresponding to the laser movement in the photodetector. In addition, sample rigidity was fitted by the gradient of force curve, with adhesion determined as the point at which the tip detached completely from the sample.

For measurements in liquid, a PFQNM-LC-A-CAL probe (Bruker AFM Probes) with a short paddle-shaped cantilever, a pre-calibrated spring constant of approximately 0.1 N m^−1^, resonance frequency of approximately 45 kHz, 65 nm radius and 17 µm tip length was used (electronic supplementary material, figure S1). This probe is suited to liquid measurements due to its gold-coated cantilever, and the small spring constant provides accurate values for biomaterials. The deflection sensitivity of the laser beam was calibrated on a glass substrate in deionized water as the RTESPA-150–30 probe oscillates at high rate (2 kHz) in air while a 0.5 kHz frequency is required for the PFQNM-LC-A-CAL probe in liquid to reduce the hydrodynamic drag. Each image in PFQNM mode was scanned at 256 × 256 points, corresponding to a 65536-pixel force-separation curves. The Derjaguin–Muller–Toporov (DMT) model was used to fit the unloading curve as this takes into account the adhesion force within the inside and outside contact areas, low adhesion and the small radii of curvature [39]. This yielded the average adhesion force (Fad), which was divided by *R* (probe radius) and 4π*R* to yield the normalized adhesion force and adhesion energy (*γ*), respectively.

To investigate the viscoelastic properties of samples, AFM-nDMA mode combined with fast force volume was used [35,36]. The PFQNM-LC-A-CAL probe was utilized in AFM-nDMA mode at room temperature and made contact with samples at a controlled force, followed by remaining in contact for 500 ms and oscillating in a sinusoidal wave at low frequencies of 100 Hz. AFM images were acquired at 4096 pixels (64 × 64 rows) over 40 min.

## 3. Results

### 3.1. Protein expression and purification

**Figure 1.**
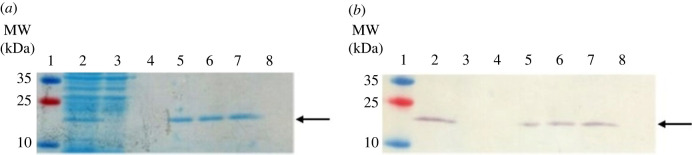
Analysis of IMAC purification of rPpolcp19k-his. (*a*). Coomassie-stained SDS-PAGE and (*b*). Western blot. Lane order in both images: Lane 1: molecular weight markers; lane 2: *E. coli* cell extract; lane 3: column flow-through; lane 4: wash fraction (containing 60 mM imidazole); lanes 5–8: elution fractions (containing 150 mM imidazole). Arrows indicate protein of the expected size.

Following expression in *E. coli* BL21 (DE3), protein extraction and purification produced yields of 1–1.5 mg of purified rPpolcp19k-his per litre of bacterial culture (figure 1). Recombinant protein yields were increased upon co-expression of GroEL-GroES chaperones, as described previously [27]. Co-overproduction of molecular chaperones [40,41] and/or folding catalysts [42] is frequently required for successful expression and folding of recombinant proteins in *E. coli*, due to saturation of the native folding machinery upon high-level production of the recombinant polypeptides. Purified proteins were dialysed into gland-like buffer (10 mM sodium acetate (pH 4.0) containing 150 mM NaCl) or seawater-like buffer (10 mM sodium phosphate (pH 8.0) containing 600 mM NaCl), prior to investigation of fibril formation.

### 3.2. Thioflavin T binding assay

**Figure 2.**
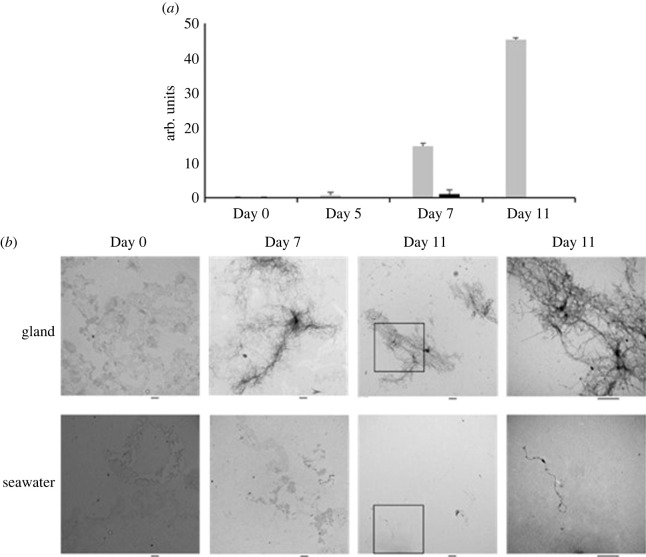
Amyloid formation by rPpolcp19k-his. (*a*). rPpolcp19k-his protein (0.5 mg ml^−1^) was incubated in cement gland-like (150 mM NaCl, pH 4.0; light columns) or seawater-like (600 mM NaCl, pH 8.0; dark columns) conditions at 25°C. Data from a Thioflavin T assay, in arbitrary units, are presented as mean ± standard deviation; *n* = 2. (*b*). TEM images of rPpolcp19k-his fibrils. Negatively stained TEM images of rPpolcp19k-his protein incubated under cement gland (upper panels) and seawater-like (lower panels) conditions at 25°C. Images are shown after 0, 7 and 11 days. The scale bar below each panel represents 500 nm. Squares in column three images represent areas shown at higher magnification in column four.

Functional amyloids are fibrillar proteins with a cross β-sheet structure which have been shown to play a role in bioadhesion in many organisms [16,43]. cp19k from *Balanus albicostatus* has previously been demonstrated to self-assemble into nanofibrils, some [44] though not all [45] of which have been shown to form amyloid. To investigate the ability of rPpolcp19k to self-assemble into amyloid fibres, the purified protein was incubated in cement gland-like (150 mM NaCl, pH 4.0) or seawater-like (600 mM NaCl, pH 8.0) conditions at 25°C. Following an initial lag, ThT fluorescence intensity increased after 7–11 days under cement gland conditions only (figure 2*a*), indicating self-assembly of rPpolcp19k-his into amyloid fibres as ThT displays enhanced fluorescence intensity when bound to the β-rich structure of amyloid fibres [38]. This initial lag period followed by a growth phase is characteristic of amyloid formation [46]. By contrast, no increase in fluorescence was observed in seawater conditions, indicating that rPpolcp19k-his does not self-assemble into amyloid under these conditions.

### 3.3. Transmission electron microscopy

To further investigate the self-assembly of rPpolcp19k-his protein into amyloid nanofibres, TEM images were captured at intervals during the assembly process. Under cement gland conditions, initial isolated fibres were visible after 5 days of incubation (not shown) while after 7 days, fibres with individual lengths of up to 1.5 µm had self-assembled into large intertwined fibrils up to 10 µm in length (figure 2*b*). By contrast to the dense network of rPpolcp19k-his fibrils observed after 11 days under cement gland conditions, only isolated rPpolcp19k-his nanofibres of up to 3 µm in length were observed after incubation in seawater conditions (figure 2*b*).

### 3.4. Morphology and mechanical properties of self-assembled rPpolcp19k-his fibres in air

**Figure 3.**
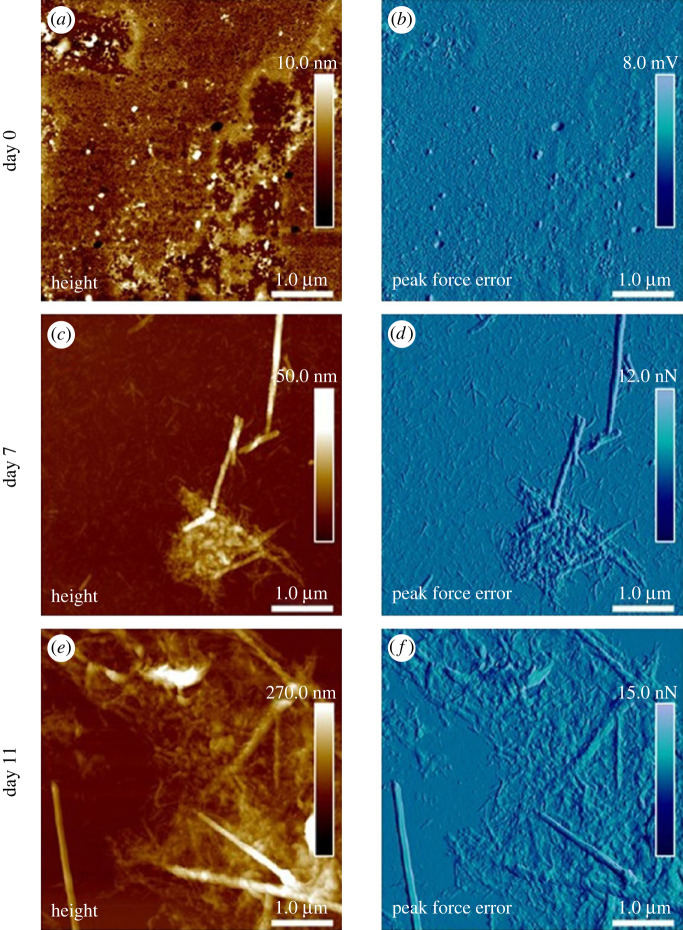
Fibril formation by rPpolcp19k-his over time, measured using PFQNM mode. (*a,c,e*). Height images. (*b,d,f*). Corresponding peak force error images. Samples were incubated under cement gland-like conditions and air-dried prior to imaging.

AFM was utilized to further investigate the structural and morphological characteristics of the rPpolcp19k-his amyloid fibrils. Tapping mode was initially employed to provide high topographic resolution at nanoscale while minimizing the force exerted on the sample surface to reduce damage to soft materials. PFQNM mode was also utilized to provide insights into the quantitative mechanical properties and topographies of the protein meshwork and fibres in air. Most of the rPpolcp19k-his protein was observed as a coating deposited on the glass substrate at day 0, with few fibres and some aggregation evident (figure 3*a,b*). After 7 days under cement gland conditions, the protein had self-assembled into a highly dense ‘meshwork’ of fibrils of up to 2000 nm in length and 50–100 nm in height, with an average length and height of single fibres of 100 and 5 nm, respectively (figure 3*c,d*). Similar fibrils were observed after 11 days, with lengths of up to 3000 nm and heights up to 100 nm, and single fibres of up to 100 nm length and 7 nm height (figure 3*e,f*). Interlacing of fibres was also evident, which became more elaborate over time. This was very different to analyses of sea star adhesive protein in our group in which increasing the NaCl concentration led to extensive surface coating by a homogeneous layer of dense *globular* nanostructures (electronic supplementary material, figure S2).

**Figure 4.**
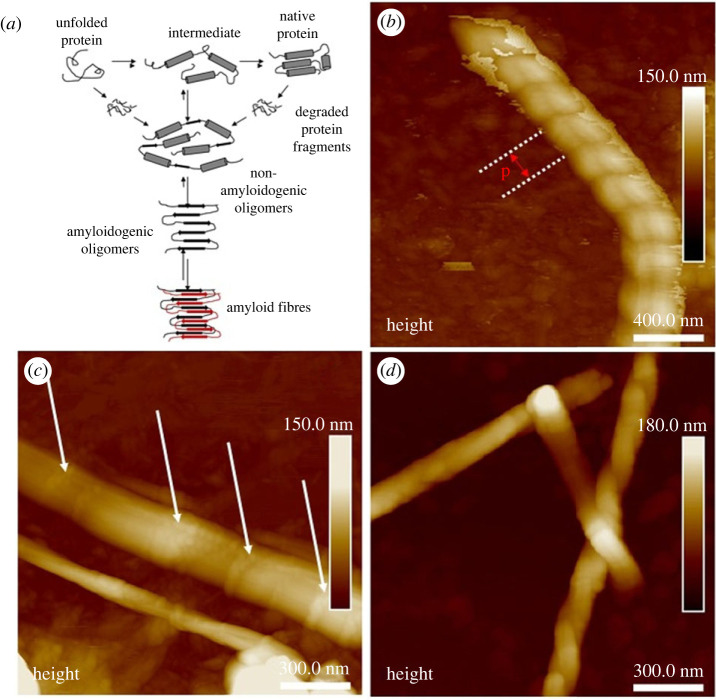
Twisted morphology of rPpolcp19k-his fibrils under cement gland-like conditions. (*a*). Model pathways of amyloid formation (based on [47]). (*b,c*). Morphology of rPpolcp19k-his fibrils at day 7. The pitch (p) of the twisted fibrils is approximately 300 nm and the arrows indicate the ‘junction’ of the bamboo-like morphology. (*d*). Morphology of rPpolcp19k-his fibrils at day 11 showing the co existence of the two morphologies. Images in (*b–d*) were captured using tapping mode.

**Figure 5.**
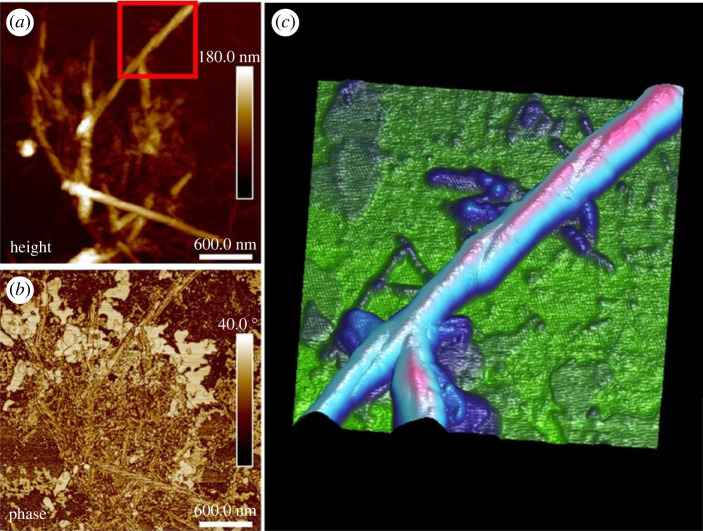
Morphology of rPpolcp19k-his fibril captured using tapping mode. Samples were incubated under cement gland-like conditions for 11 days, followed by air-drying prior to measurement. (*a,b*). Height and phase images, respectively, of rPpolcp19k-his; image size is 3 × 3 µm. (*c*) Three-dimensional rendering image of rPpolcp19k-his with phase data superimposed on topography from panel (*a*). Area shown is red square highlighted in (*a*). Image size is 1 × 1 µm.

To enhance image quality and obtain greater detail of fibres, a tip with 8 nm radius was used in tapping mode over a few micrometres. The rPpolcp19k-his fibrils were found to be arranged in parallel, with individual oligomers running perpendicular to the fibril axis (figure 4*a*). At day 7 under cement gland-like conditions, fibrils had assembled into a structure consisting of several amyloid oligomers organized into a distinctive bamboo-like pattern, with fibrils reaching approximately 50 nm in height and ranging from 50 to 250 nm in width (figure 4*b,c*). The bamboo-like and twisted fibril structures were also evident on day 11 as seen in figure 4*d*, with amyloid fibrils now overlaid on top of each other into a meshed appearance (figure 3*c*,*e*; electronic supplementary material, figure S3*a*). The bamboo-like fibril structure had a height of 12 nm, width of 170 nm and length of 950 nm, while the twisted fibril structure was much larger with respective dimensions of 55, 160 and 1570 nm. This distinctive twisting of fibrils was also evident in samples analysed in standard tapping mode after incubation for 11 days under gland-like conditions at pH 4.0 (figure 5).

**Figure 6.**
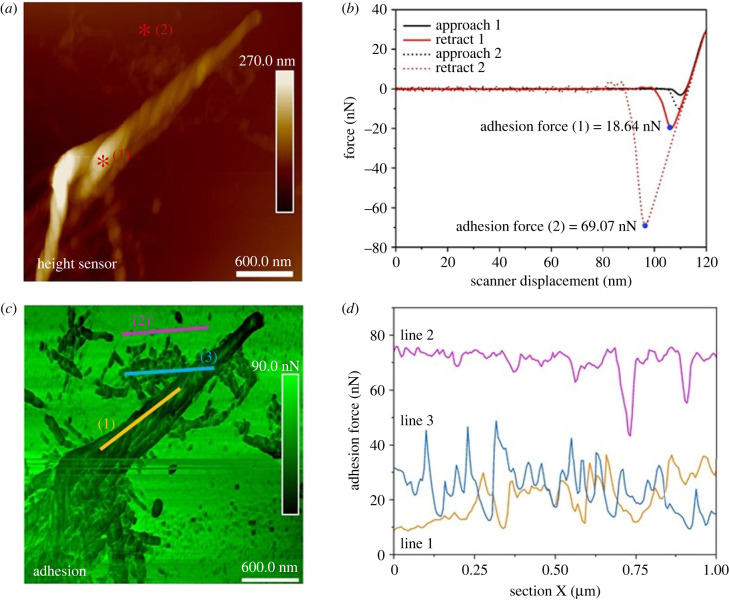
(*a*). Morphology of rPpolcp19k-his fibrils developed over 11 days under gland-like conditions. (*b*). Force curves (black lines: approach; red lines: retract) versus scanner displacement corresponding to the red asterisks at points 1 (fibril) and 2 (meshwork) on the height image in (*a*). (*c*) Adhesion image corresponding to image (*a*). (*d*) Adhesion force determined for lines 1, 2, 3 shown in image (*c*). Measurements were made in air and data were fitted to the DMT model using Nanoscope Analysis 2.0 (*R*^2^ = 0.99).

Imaging of the same samples in PFQNM mode to investigate their viscoelasticity revealed a modulus of 2.5 ± 0.8 GPa and adhesion of 20 ± 7.5 nN, determined in air for the fibrils on day 11 (electronic supplementary material, figure S3) using the RTESPA-150–30 probe. The twisted fibrils were determined to reach up to 200 nm in height, with an adhesive force approximately three times less than in the surrounding meshwork (figure 6).

### 3.5. Elasticity and adhesion of rPpolcp19k-his in liquid

**Figure 7.**
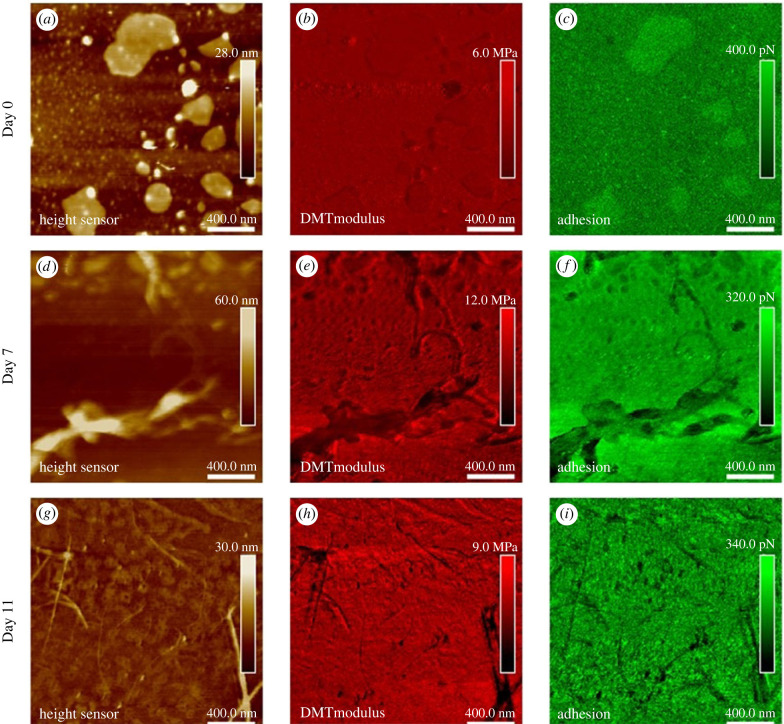
PFQNM AFM analysis of rPpolcp19k-his fibril formation in deionized water. Images were captured after protein incubation in cement gland-like conditions for 0 (*a–c*), 7 (*d–f*) or 11 (*g–i*) days prior to analysis. (*a,d,g*) height sensor; (*b,e,h*) modulus; and (*c,f,i*) adhesion images. Scale bars are 400 nm and scan size is 2 × 2 µm.

Further investigation of the nanomechanical properties of the rPpolcp19k-his amyloid fibrils was carried out in liquid measurement, using PFQNM and nDMA, to gain more physiologically relevant information on the morphology and rigidity of the fibres. Measurements were performed in Milli-Q water in order to focus on the instinctive adhesion ability of the protein and avoid the influence of electrostatic attraction between charged proteins and glass substrates [48]. The mechanical properties of the fibres formed under cement gland conditions and measured using PFQNM mode are shown in figure 7. The applied force was controlled at 700 pN to avoid damaging the sample and a soft probe PFQNM-LC-A-CAL was used with a 0.1 N m^−1^ spring constant. The majority of protein formed large zones on day 0 while some fibrils were observed after 7 and 11 days under cement gland conditions (figure 7*a,d,g*). The fibrils formed in water were smaller than those formed in air (electronic supplementary material, figure S4).

The modulus and adhesion of rPpolcp19k-his in liquid conditions using PFQNM AFM were mapped on days 0, 7 and 11 as shown in figure 7. A single force curve was extracted from figure 7*g,h,i* and calculated using a DMT model (see electronic supplementary material, figure S5) to give a stiffness of 3.18 and 6.01 MPa for the approach and retract curves, respectively. Differences in the stiffness between approach and retract curves arise mainly from the inherent viscosity and strong adhesion of materials. Calculated modulus and adhesion values were fitted with a Gaussian model (electronic supplementary material, figure S6) and are close to those measured for the commercial mussel adhesive protein mixture Cell-Tak™ in this study (electronic supplementary material, figure S7), indicating that the protein is strongly adhesive under both cement gland and seawater conditions, in agreement with the adhesion values observed over days 0–7–11 (table 1). No significant difference was found in approach, retract modulus or adhesion of rPpolcp19k-his during fibre formation over days 0, 7 and 11 samples at pH4.0.

**Table 1.** Mechanical properties (modulus and adhesion) of rPpolcp19k-his proteins incubated in gland-like (pH 4.0, 150 mM NaCl) or seawater-like (pH 8.0, 600 mM NaCl) conditions, measured by PFQNM or nDMA in liquid. Figure 6 (single value extraction) and electronic supplementary material, figure S6 (determination of statistical calculations) for details on the DMT model for measuring modulus.

	conditions	method	DMT modulus (MPa)		adhesion
			approach	retract	
cp19-pH4.0-day 0	liquid	PFQNM	2.7 ± 0.7	5.2 ± 1.2	150 ± 30 pN
cp19-pH4.0-day 7	liquid	PFQNM	2.6 ± 0.9	5.8 ± 1.2	110 ± 33 pN
cp19-pH4.0-day 11	liquid	PFQNM	3.8 ± 0.5	6.0 ± 0.8	135 ± 48 pN
cp19-pH8.0-day 7	liquid	PFQNM	3.0 ± 0.5	5.3 ± 0.9	107 ± 43 pN
cp19-pH8.0-day 11	liquid	PFQNM	2.4 ± 0.2	3.7 ± 0.7	89 ± 37 pN

Under seawater conditions, rPpolcp19k-his failed to self-assemble into amyloid fibrils after 7 days, with only a few isolated fibres observed on day 11 (electronic supplementary material, figure S8), while large protein aggregates which deposited on the substrate were observed after 7 and 11 days.

Adhesion values were extracted from the negative force in the retract curve under PFQNM measurements (see electronic supplementary material, figure S9 and table 1). Upon normalization, the adhesion of rPpolcp19k-his proteins on days 0, 7 and 11 under cement gland conditions was 2.31 ± 0.46, 1.69 ± 0.51 and 2.08 ± 0.74 mN m^−1^, respectively, with corresponding adhesion energies of 0.18 ± 0.03, 0.13 ± 0.04 and 0.16 ± 0.05 mJ m^−2^. In seawater conditions, the normalized adhesion of rPpolcp19k-his on days 7 and 11 was 1.65 ± 0.66 and 1.37 ± 0.56 mN m^−1^, respectively, and the adhesion energy was 0.13 ± 0.05 and 0.11 ± 0.04 mJ m^−2^, respectively. Therefore, while the protein exhibited slightly higher adhesion values in gland than in seawater conditions, no change in adhesion was detectable upon fibre formation in gland-like conditions.

Pocius [49] notes that, for good adhesion to take place, the adhesive and the surface must come into intimate contact, which requires the adhesive to spread spontaneously over the surface to maximize interfacial contact, whereas fibre formation in the rPpolcp19k-his sample at days 7 and 11 is expected to result in reduced contact with, and coverage of, the surface. Notably in this study, though adhesion values did not change significantly for the rPpolcp19k-his protein during fibre formation, the values were similar to those measured for Cell-Tak™ (electronic supplementary material, figure S7). Cell-Tak™ is commonly used in comparative investigations of adhesiveness [48,50–53] and two phases were detected in its adhesion and modulus in this study, depending on meshwork formation: when Cell-Tak™ formed particles, these exhibited adhesion of 215.74 ± 135.38 pN with a Young's modulus of 0.80 ± 0.12 MPa, whereas adhesion of the meshwork was significantly lower at 62.22 ± 13.10 pN, with an increase in Young's modulus to 2.96 ± 1.29 MPa (electronic supplementary material, figure S7). The normalized adhesion and adhesion energy of Cell-Tak-derived particles were 3.32 ± 2.01 and 0.26 ± 0.16 mJ m^−2^, respectively, compared with 0.95 ± 0.10 and 0.07 ± 0.01 mJ m^−2^ for the protein meshwork. This was higher but not substantially different from the values determined for rPpolcp19k-his, in both gland and seawater conditions at all three time points (table 1), i.e. after it had formed amyloid fibres, but also in its non-assembled protein coating form.

### 3.6. Viscoelasticity of rPpolcp19k-his

**Figure 8.**
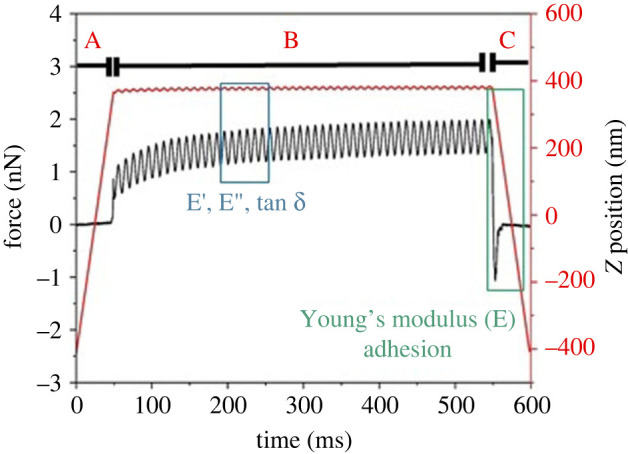
AFM-nDMA analysis of rPpolcp19k-his incubated for 11 days under gland conditions (pH 4.0), at day 11. The red line represents the Z position trajectory (right y-axis) and the black line the spectroscopy force (left y-axis). After the tip is brought into contact with the sample, a preload is applied (indicated by A), followed by modulation of the frequency at 100 Hz B, from which values for *E*′, *E*′′ and tan δ are calculated, and retraction of the tip from the sample C.

Interaction between the AFM tip and the rPpolcp19k-his protein occurred at a low, well-controlled force of 1 nN (figure 8, segment A), followed by modulation of the frequency to oscillate the tip while in contact with the sample surface (figure 8, segment B). In AFM-nDMA, this occurs at rheologically relevant frequencies in the range of 0.1 to 100 Hz, unlike traditional AFM measurements that are performed at kHz and MHz. As this necessitates additional measurement time for AFM-nDMA, PFQNM is initially used to generate maps of the modulus and adhesion of a sample relatively quickly and with high resolution, followed by the use of AFM-nDMA to study the viscoelastic properties of specific regions in more detail.

Measurements were performed at 100 Hz in this work (figure 8, segment B) to ensure reasonable data acquisition times. This allowed the amplitude and phase of Z displacement (Z scanner) and probe deflection to be extracted, and E′, E′′ and tan *δ* to be calculated using the equation of Pittenger *et al.* [36]. Finally, the adhesion and Young's modulus were measured in the retract curve (figure 8, segment C) as the tip was pulled away from the sample.

**Figure 9.**
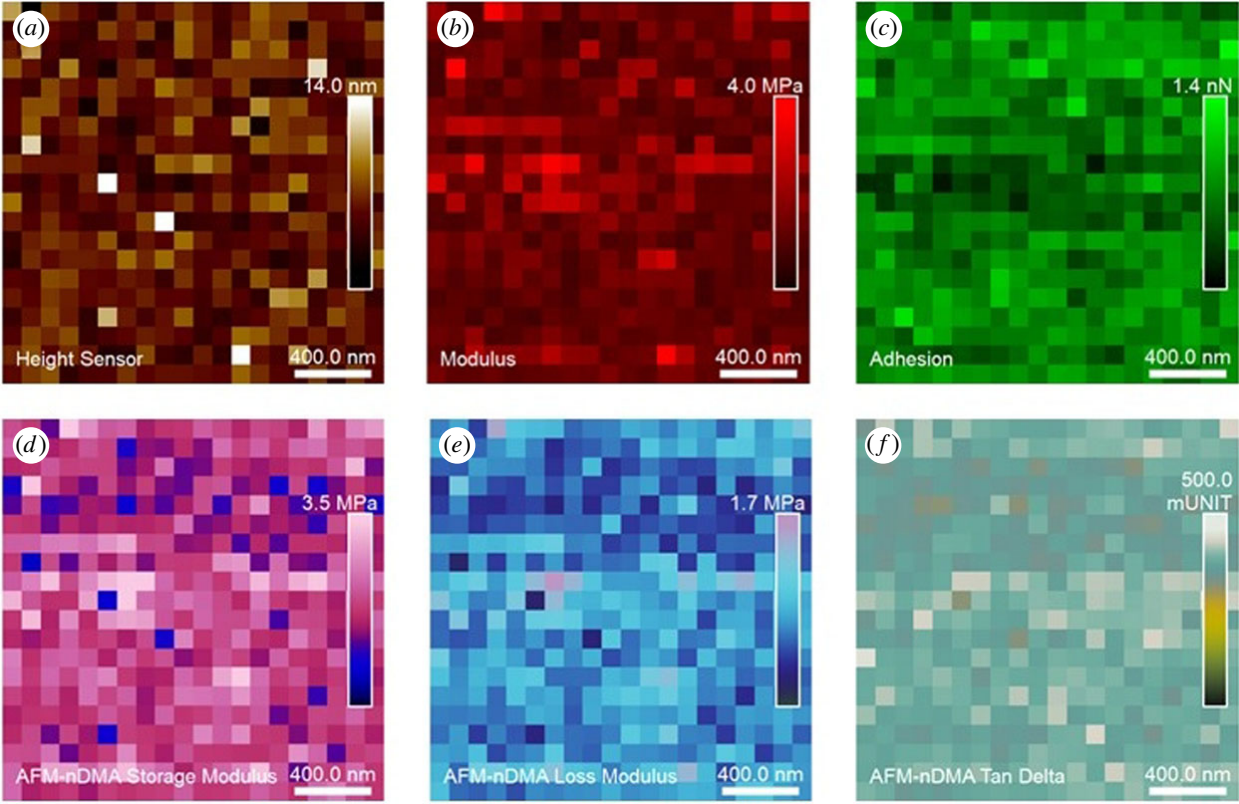
AFM analysis of rPpolcp19k-his protein in AFM-nDMA mode. Panels indicate (*a*) height; (*b*) modulus; (*c*) adhesion; (*d*) storage modulus; (*e*) loss modulus; (*f*) tan δ. The modulus and adhesion panels were computed from segment C indicated in figure 8; *E*′, *E*′′ and tan δ were computed from segment B indicated in figure 8. Samples were incubated under cement gland-like conditions for 11 days prior to analysis in deionized water. Scan sizes are at 2 × 2 µm with 400 pixels.

The viscoelastic properties of the rPpolcp19k protein fibres were quantified after 11 days under cement gland conditions, using measurement in deionized water. Height, modulus, adhesion, storage modulus, loss modulus and tan δ data images are shown in figure 9. Values of *E*′ (elasticity) = 2.8 ± 0.5 MPa, E′′ (viscosity) = 1.2 ± 0.4 MPa and tan δ (relative degree of energy dissipation) = 0.37 ± 0.03 were calculated. The measurements incidate that rPpolcp19k-his exhibits both elasticity and viscosity, with the former dominating its mechanical properties, while the tan *δ* value indicates that the rPpolcp19k-his protein after day 11 occurs in a soft solid form.

A Young's modulus of 1.2 ± 0.6 MPa and adhesion of 1.1 ± 0.5 nN were measured for the fibres formed under gland (pH 4.0) conditions after 11 days in AFM-nDMA mode (figure 8, segment C) by fast force volume, with the former considerably lower than that measured with PFQNM, and the adhesion markedly higher than the 110–150 pN range obtained using PFQNM with the same PFQNM-LC-A-CAL probe (table 1). As the PFQNM mode works at 2 kHz (with a very short interaction time of approximately 1 ms) whereas the cantilever oscillates at 100 Hz in AFM-nDMA (with an interaction time of approximately 500 ms during the hold segment), the longer contact time between tip and sample in AFM-nDMA leads to deeper indenting of the tip. In addition, the viscosity of the rPpolcp19k-his protein is not considered in PFQNM, whereas in AFM-nDMA the E′′ value considers the loss energy of rPpolcp19k-his, resulting in a lower rigidity modulus that is closer to the ‘real’, *in situ* value of the protein than that resulting from PFQNM. Thus, AFM-nDMA is expected to provide more accurate measurement of viscoelastic properties of materials, including biomaterials, pharmaceuticals and adhesives, as well as living cells in their native biophysical conditions.

## 4. Discussion

### 4.1. Adhesion in marine species

Amyloid fibrils form a substantial proportion of barnacle cement, with up to 30% of the ‘nanofibrillar matrices' comprising the bulk cement estimated to be formed by these fibrils [16]. This study characterizes the conditions under which a protein component of the *P. pollicipes* bulk cement undergoes amyloid fibre formation, which is integral to understanding barnacle adhesion and the role of fibres in barnacle cement. The barnacle adhesive complex hardens into a cement over many hours [4] via processes in the constituent proteins which appear to include hydrogen bonding, hydrophobic interactions and protein folding [17,54,55], with evidence that at least some of these changes are triggered by changes in pH and the ionic environment (this study, [48]). Functional ‘units’ of β-sheet-rich amyloid fibres have been reported in at least two constituent proteins from barnacle adhesives: cp19 kDa (this study; and *Balanus amphitrite* Bacp19k [16]) and potentially other proteins possessing amyloidogenic motifs, e.g. Mrcp52k from *Megabalanus rosa* [54]. The cp19k protein from *P. pollicipes* has the highest predicted composition of β-sheets among its homologues in a number of barnacle species [24], while fibres formed from the homologous protein in *B. albicostatus* (rBalcp19k) are highly resistant to degradation [48], which has obvious advantages for the organism.

### 4.2. Fibre formation by rPpolcp19k-his

The cp19k protein from barnacles has been suggested to occur at the interface between the bulk cement and the external substrata, and to play a critical role in adhesion of the animal to its underwater substrate [18,48]. While a number of studies have assigned it an adhesive role [3,18], characterization of its molecular structure has instead identified its ability to form β amyloids and to self-assemble into nanofibres under differing conditions (this study, [19,48]). The dependence of fibre formation on physico-chemical conditions remains unclear, however, and fibres formed by rBalcp19k have been reported to *not* exhibit amyloid properties or to increase in β-sheet content during assembly [48].

The *P. pollicipes* 19 kDa protein was demonstrated to self-assemble into amyloid (i.e. ThT-positive) fibres under low pH and low ionic (gland-like) conditions in the present study. rBalcp19k, with a similar pI to the present protein, self-assembled into fibres under similar low pH and low ionic conditions [48]—after a Cysteine-substituted variant was originally found to form nanofibrils under ‘seawater’ rather than ‘gland’ conditions [44]. Unlike in the present study, however, rBalcp19k fibres were not amyloidogenic and they became ‘curled’ and entangled into balled aggregates [48] rather than forming the interlaced and twisted fibres observed in the present work.

Formation of fibres at low pH conditions which mimic those in the gland appears to risk the premature formation of a mature adhesive inside the organism's own gland—particularly as all the protein components are produced inside the same giant cell in barnacles, unlike in other marine adhesive organisms [21]. Barnacle glues are slow to harden, however, and it is likely that the cement undergoes cross-linking and slowly begins to cure in the canals, which may avoid blocking them during secretion [45]. Fibrillogenesis has been described in a variety of cell types, from eukaryotes to bacteria, with the latter devoting significant molecular machinery to curli fibre production, to broaden the ‘repertoire’ of structures available to them [56]. As it is potentially catastrophic in the wrong circumstances, however, fibril formation is rigidly controlled using cell machinery, enzymatic checks and, based on the present work, environmental control of the extruded proteins. Meanwhile, the adhesives of barnacle larvae are relatively fast-setting, presumably because the larvae have a very short time window in which to settle and metamorphose, though even these require up to 15 h to cure [57].

### 4.3. Adhesion analysis of rPpolcp19k-his

Liang and co-workers reported stronger adhesion in unassembled rBalcp19k at lower pH values (pH 3.6, 5.0, 8.0, 9.9: 0.88, 0.65, 0.32, 0.12 mN m^−1^), which was comparable to Cell-Tak™ under the same conditions (1.54, 0.82, 0.23, 0.12 mN m^−1^, respectively) [48]. Conversely, their pre-assembled fibres exhibited considerably higher adhesion at pH 8.0 or in seawater conditions (6.01–7.25 mN m^−1^) compared with pH 3.6 or 5.0 (2.66–3.80 mN m^−1^), which they hypothesized to indicate that relatively non-adhesive fibres may be formed in the low-pH-low-ionic-strength setting of the animal gland, followed by an increase in adhesion upon extrusion to the higher-pH-higher-ionic-strength seawater environment. By contrast, the rPpolcp19k-his protein exhibited similar adhesion in samples lacking fibres and throughout extensive fibre self-assembly over 11 days in the present study (1.69–2.31 mN m^−1^), which was comparable to Cell-Tak™ (meshwork: 3.32 mN m^−1^; particles: 0.95 mN m^−1^), as well as in seawater and cement gland conditions. While rBalcp19k differs fundamentally from *P. pollicipes* cp19k in forming non-amyloid fibres [48], rPpolcp19k fibres may—similar to rBalcp19k—function to concentrate the adhesive protein at the surface interface upon secretion and prevent its over-dilution prior to adherence. It has also been proposed to ‘prime’ the initial stage of cement adhesion, coupling with the more highly abundant cp68k [55], which has a hydrophobic *N*-terminal region that may function in interacting between cement proteins and the water layer [17], though it may also have roles in surface coverage, providing additional cohesive strength or increasing degradation resistance in the barnacle cement. Additionally, the lack of DOPA in the *P. pollicipes* cp19k protein, unlike Cell-Tak™, lends the barnacle protein to recombinant expression in heterologous hosts such as *E. coli* which are unable to carry out this post-translational modification, whereas Cell-Tak™ must be purified using strong acids from the native animals.

The large difference in adhesion values measured for rPpolcp19k-his using PFQNM (89 pN) and AFM-nDMA (1.1 nN) arises from the frequency difference between the analytical methods, with the cantilever oscillating at kHz in the former mode but at 100 Hz in AFM-nDMA. In addition, consideration of the viscosity of the protein in AFM-nDMA analyses results in a lower rigidity modulus (electronic supplementary material, figure S5). Combination of the two approaches allowed classical measurements of adhesion, but also deformation, indentation and energy dissipation, to be extracted in approximately 2 h (for a 128 × 128 pixel acquisition). The measured values are similar to those reported by Phang *et al.* [58], who measured adhesion in barnacle larval bulk adhesive of pico to nano newtons—and described how the bulk protein unfolds by virtue of ‘sacrificial bonds’ but then amazingly, ‘reforms’ once the AFM force relaxes.

It has been speculated that an STGA-rich block in *B. albicostatus* cp19k, which alternates with a block containing charged and hydrophobic amino acids and is homologous to the assembly sequences of silk proteins [19], is likely to be the self-assembly motif [48]. While biomimetic peptides based on both sequence types could self-organize into amyloid-like nanofibrils, only the STGA-rich sequences could seed further protein self-assembly. Inspection of rPpolcp19k-his sequence identified a similar block copolymer-type structure, in which four STGA-rich blocks also alternate with five charged/hydrophobic domains (electronic supplementary material, figure S10).

### 4.4. Viscoelasticity analysis of rPpolcp19k-his

The combination of PFQNM and AFM-nDMA approaches enabled comprehensive characterization of rPpolcp19k-his in liquid conditions: PFQNM provided topographic images and roughness measurements in air and water, while AFM-nDMA mode measured the viscoelastic properties of the protein, determined by storage modulus, loss modulus and tan δ. Importantly, this combination of approaches allowed the properties of the assembled fibres to be determined in conditions that are closer to their native environment [35,36] and provides a more detailed, in-depth understanding of soft materials than simply measuring their mechanical properties. AFM-nDMA, in particular, takes advantage of the exquisite force sensitivity, small contact radius and nanoscale indentation depth of AFM to provide dynamic mechanical analysis and quantitative viscoelastic analysis down to 10 nm spatial resolution [35,36].

Analysis of the surface-coated rPpolcp19k-his indicated that, while it exhibited both elasticity and viscosity, its elasticity dominated its mechanical properties. The measured tan δ value of 0.37 indicates that the protein is a soft and viscoelastic material [59]. While fibre morphology might not be expected to differ greatly under dry or wet measurement conditions, the presence of water can have a considerable impact on the mechanical properties of the fibres: wet conditions are more likely to maintain the proteins in their natural state, while measurement under dried conditions can overestimate properties such as rigidity or adhesion by orders of magnitude. Our approach of making measurements in humid conditions using specialized probes is particularly relevant since bulk cements from barnacles have been shown to be ‘highly hydrated’ [16].

Amyloidogenic peptides assemble into quaternary structures which are remarkably stable over time once fully formed and exhibit diverse morphologies depending on the incubation conditions [60]. The advantages of fibrils include their strength and stiffness, surface deposition, stability and resistance to degradation [61]. In the future, new functionalized, amyloid fibril-based materials could be envisaged, to form structures ranging from nanotubes to lamellar crystals, or reversible and pH-dependent hydrogels with diverse applications in cell adhesion, drug delivery or tissue engineering [62–64].

## 5. Conclusion

The *P. pollicipes* cement protein formed stable amyloid fibres in conditions that mimic the native organism's cement gland. The fibres self-assembled into large intertwined fibrils after 7–11 days, with a distinctive twisting of the fibrils evident after 11 days. The mechanical and viscoelastic properties of the fibres at the nanoscale revealed that the barnacle protein is a soft and viscoelastic material, with adhesion strength, in both unassembled and fibre forms, comparable to that of the commercial adhesive Cell-Tak. This work provides an in-depth understanding of the behaviour of the protein and its self-assembled fibres in their natural environment and may be used to inform the design of amyloid fibril-based biomaterials or bioadhesives for diverse applications.

## Data Availability

Datasets used for the study are available at https://doi.org/10.6084/m9.figshare.c.6736299. The data are provided in electronic supplementary material [65].
